# AIM2 inhibits colorectal cancer cell proliferation and migration through suppression of Gli1

**DOI:** 10.18632/aging.202226

**Published:** 2020-12-03

**Authors:** Menglin Xu, Junfeng Wang, Haoran Li, Zhengrong Zhang, Zhengwu Cheng

**Affiliations:** 1 Department of Oncology, The First Affiliated Hospital of Wannan Medical College, Wuhu 241000, China; 2 Department of Gastrointestinal Surgery, The First Affiliated Hospital of Wannan Medical College, Wuhu 241000, China

**Keywords:** AIM2, proliferation, migration, Gli1, colorectal cancer

## Abstract

Colorectal cancer (CRC) is a common malignant tumor and is one of the leading causes of cancer-related deaths worldwide. Absent in melanoma 2 (AIM2), as a member of the pyrin-HIN family proteins, plays contentious roles in different types of cancers. In the present work, we provide evidence that AIM2 was commonly downregulated in human CRC and loss of AIM2 significantly correlated with tumor size, depth of invasion, lymph node metastasis (LNM) and TNM (Tumor, Node, Metastases) stage in patients suffering from CRC. AIM2 knockdown promoted CRC cell proliferation, migration and epithelial-mesenchymal transition (EMT) progress, whereas AIM2 overexpression did the opposite. AIM2 inhibited glioma-associated oncogene-1 (Gli1) expression through Smoothened homolog (SMO)-independent pathway and regulated CRC cell proliferation and migration in a Gli1-dependent manner. Moreover, AIM2 could modulate Protein kinase B (AKT)/mechanistic target of rapamycin (mTOR) signaling pathway and the increased Gli1 expression and EMT progress induced by AIM2 depletion was reversed after incubation with AKT inhibitor Ly294002 in CRC cells. In conclusion, our results define AIM2 as a novel regulator of Gli1 in CRC cell growth and metastasis, and suggest that the AIM2/AKT/mTOR/Gli1 signaling axis may serve as a potential target for treatment of CRC.

## INTRODUCTION

Colorectal cancer (CRC) is a common malignant tumor and is one of the leading causes of cancer-related deaths worldwide [1, 2], with annually rising incidence rates and high mortality [3]. CRC patients diagnosed at early stage have a favorable prognosis, with a 5-year survival rate of 70% to 90% [4]. Unfortunately, some CRC patients are diagnosed at advanced or metastatic stages, with a low 5-year survival rate [5]. Therefore, it is of great clinical importance to identify novel targets and to establish timely and appropriate treatment to prevent CRC progression.

Absent in melanoma 2 (AIM2), as a member of the pyrin-HIN family proteins, was originally isolated from healthy melanocytes [6]. AIM2 senses cytosolic double-stranded DNA (dsDNA) and binds to its inflammasome adaptor, apoptosis-associated speck-like protein containing a carboxy-terminal CARD (ASC), to activate the inflammasome [7, 8]. In addition to its role in inflammasome activation, AIM2 has recently been described as both a oncogene and tumor suppressor in different types of cancers. In oral squamous cell carcinoma (OSCC), AIM2 was overexpressed and ectopic AIM2 expression enhanced OSCC cell proliferation and prevented cell apoptosis [9]. Furthermore, AIM2 was highly expressed in non-small cell lung cancer, and its overexpression facilitated cell growth and predicted poor survival of patients [10, 11]. On the other hand, excessive AIM2 expression suppressed tumor growth in breast carcinoma [12]. AIM2 was also reported to be down-regulated in hepatocellular carcinoma and loss of AIM2 expression contributed to hepatocarcinoma tumorigenesis and metastasis [13, 14]. Moreover, several recent studies demonstrated that lack of AIM2 expression exhibited oncogenic properties and was closely associated with poor outcome in CRC [15–18], but the precise functional roles and underling mechanisms of AIM2 in CRC remain to be further explored.

The Hedgehog (Hh) signaling pathway is considered to play an essential role in cancer development and progression, including CRC [19–21]. The Hh ligand, including Sonic Hedgehog (SHh), Indian Hegehog (IHh), and Desert Hedgehog (DHh), binds to its receptor, protein patched homolog (PTCH), leading to the release of the smoothened (SMO). Released SMO then activates glioma-associated oncogene 1 (Gli1) by blocking its inhibitory partner, suppressor of fused homolog (SUFU). The SMO-dependent Gli1 activation is termed as the canonical Hh pathway [22]. The Gli1 protein, as a key transcriptional factor of Hh pathway, can also be activated by protein kinase B (AKT), extracellular signal regulated kinase (ERK) and mammalian target of rapamycin (mTOR) in a SMO-independent manner, which is known as non-canonical Hh pathway [22, 23]. Although the classical Hh pathway has been well studied, how Gli1 is regulated by SMO-independent way remains elusive.

In this study, we discovered a reduced expression and anti-tumor properties of AIM2 in CRC. Moreover, our results provided new mechanistic insights into the crucial roles of AIM2 in suppressing Gli1 through regulating AKT/mTOR pathway, which reveals a possible implication for new approaches to CRC therapy.

## RESULTS

### AIM2 expression is significantly decreased in CRC tissues

**Figure 1:**
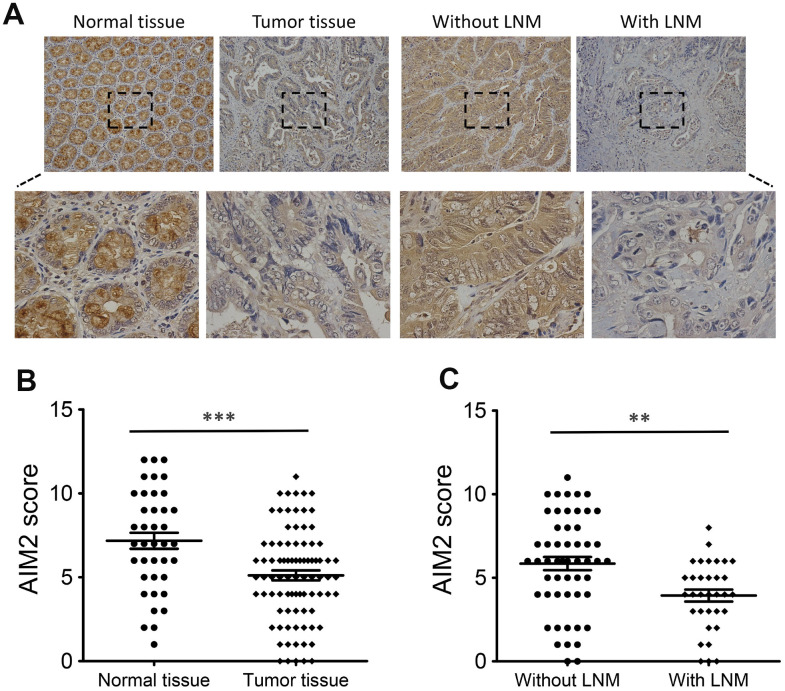
**Expression of AIM2 protein in human CRC tissues.** (**A**) IHC staining of AIM2 in CRC tumor tissues and surrounding normal tissues. (**B**) Scatter plot analysis of AIM2 IHC scores in CRC tumor tissues (n=86) and normal tissues (n=39). (**C**) Scatter plot analysis of AIM2 IHC scores in CRC tumor tissues with (n=31) or without LNM (n=55). **P<0.01, ***P<0.001, based on a two-tailed unpaired Student’s t-test.

To further explore the anti-carcinogenic roles of AIM2 in colorectal tumorigenesis, we firstly analyzed AIM2 protein expression in human CRC tissues and para-cancer normal tissues using Immunohistochemistry (IHC) analysis. IHC staining revealed that high AIM2 protein expression was seen in 59.3% (51/86) of CRC tumor samples examined, whereas 79.5% (31/39) of normal tissues showed strong AIM2 signal (Figure 1A, 1B, Supplementary Figure 1 and Table 1). The difference was statistically significant (P < 0.001, Figure 1B). Further analysis showed that AIM2 level in human CRC tissues with LNM was lower than those released from LNM (P < 0.01, Figure 1A, 1C).

**Table 1:** Correlation of AIM2 protein expression with clinic-pathological factors in 86 patients with CRC.

**Clinic parameters**	**Total**	**AIM2 expression**		**χ^2^**	***P* value**
		**None or low**	**High**		
**Total**	86	35 (40.7%)	51 (59.3%)		
**Age (years)**					
<65	35	13 (37.1%)	22 (62.9%)	0.309	0.578
>=65	51	22 (43.1%)	29 (56.9%)
**Gender**					
Male	58	22 (37.9%)	36 (62.1%)	0.565	0.452
Female	28	13 (46.4%)	15 (53.6%)
**Tumor size**					
<5cm	47	14 (29.8%)	33 (70.2%)	5.112	0.024*
>=5cm	39	21 (53.8%)	18 (46.2%)
**Tumor location**					
Left-sided colon	28	13 (46.4%)	15 (53.6%)	0.582	0.748
Right-sided colon	27	10 (37.0%)	17 (63.0%)
Rectum	31	12 (38.7%)	19 (61.3%)
**Depth of invasion**					
T1-2	16	2 (12.5%)	14 (87.5%)	6.476	0.011*
T3-4	70	33 (47.1%)	37 (52.9%)
**Lymph node metastasis**					
Yes	31	20 (64.5%)	11 (35.5%)	11.394	0.001**
No	55	15 (27.3%)	40 (72.7%)
**TNM stage**					
I	13	0 (0%)	13 (100.0%)	13.699	0.003**
II	39	15 (38.5%)	24 (61.5%)
III	32	19 (59.4%)	13 (40.6%)
IV	2	1 (50.0%)	1 (50.0%)

Next, we investigated the clinical relevance of AIM2 expression in 86 CRC tumor tissues. Chi-square test analysis showed that reduced AIM2 in CRC was significantly associated with tumor size (P=0.024, Table 1), depth of invasion (P=0.011, Table 1), LNM (P=0.001, Table 1) and TNM stage (P=0.003, Table 1). However, there was no significant correlation between AIM2 protein level and other clinicopathological factors such as age, gender and tumor location (P > 0.05, Table 1).

Collectively, these results suggested that AIM2 is down-regulated in the development of CRC and loss of AIM2 correlates with some unfavorable clinicopathological features of CRC patients.

### AIM2 expression in CRC cell lines

**Figure 2:**
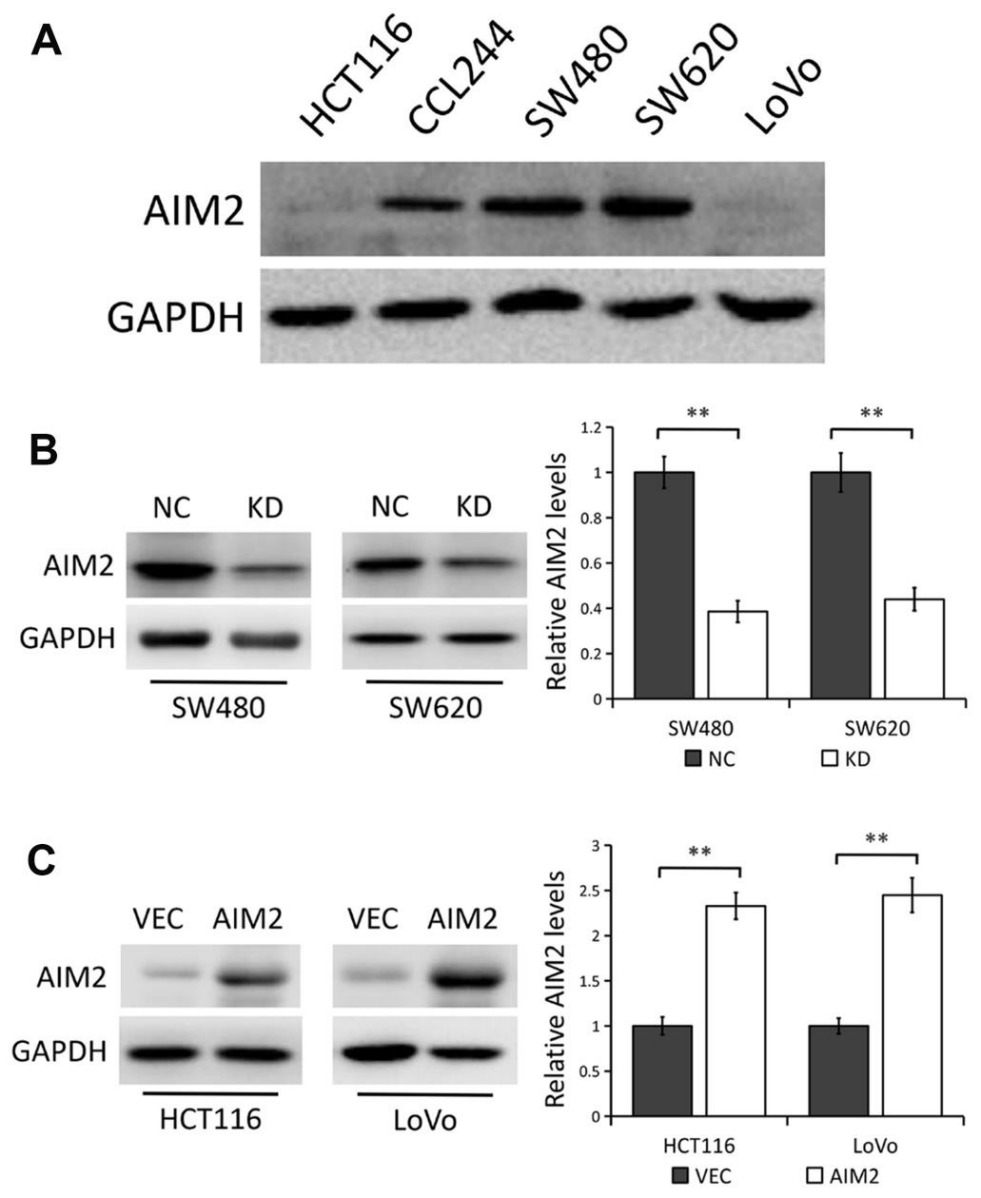
**AIM2 protein expression in human CRC cell lines.** (**A**) Western blots of AIM2 protein in five human CRC cell lines (HCT116, CCL244, SW480, SW620 and LoVo). GAPDH as a loading control. (**B**) Western blots of AIM2 protein in SW480 and SW620 cells stably transfected with control-shRNA (NC) or shRNA against AIM2 (KD). GAPDH as a loading control. Each experiment was performed at least triplicate and the bands were quantified and presented as the mean±SEM. (**C**) Western blots of AIM2 protein in HCT116 and LoVo cells stably transfected with empty vector (VEC) or plasmid encoding human AIM2 (AIM2). GAPDH as a loading control. Each experiment was performed at least triplicate and the bands were quantified and presented as the mean±SEM. **P<0.01, based on a two-tailed paired Student’s t-test.

We first detected the levels of AIM2 protein in five different human CRC cell lines (HCT116, CCL244, SW480, SW620 and LoVo) by Western blot analysis. Compared with the low AIM2 protein expression in HCT116 and LoVo cell lines, AIM2 levels in SW480 and SW620 were much higher (Figure 2A).

Thus, in the subsequent experiments, a lentivirus vector-based shRNA technique was used to stably knocked down AIM2 expression in SW480 and SW620 cells with relatively high endogenous AIM2 level, while plasmids encoding human AIM2 or corresponding empty vector were introduced into HCT116 and LoVo cells which express relatively low endogenous AIM2. Results from Western blot analysis showed that AIM2 protein level was significantly lower in SW480 and SW620 cells stably transfected with AIM2-targeting shRNA (KD) than those transfected with control-shRNA (NC) (P<0.01, Figure 2B), while AIM2 protein expression was greatly increased in cells stably transfected with human AIM2 (AIM2) compared to cells transfected with empty vector (VEC) (P<0.01, Figure 2C). Furthermore, we used an AIM2-specific siRNA to knock down AIM2 expression in SW620 cell line and the knockdown efficiency was confirmed by Western blot analysis (Supplementary Figure 2A).

### AIM2 plays anti-carcinogenic roles in CRC

**Figure 3:**
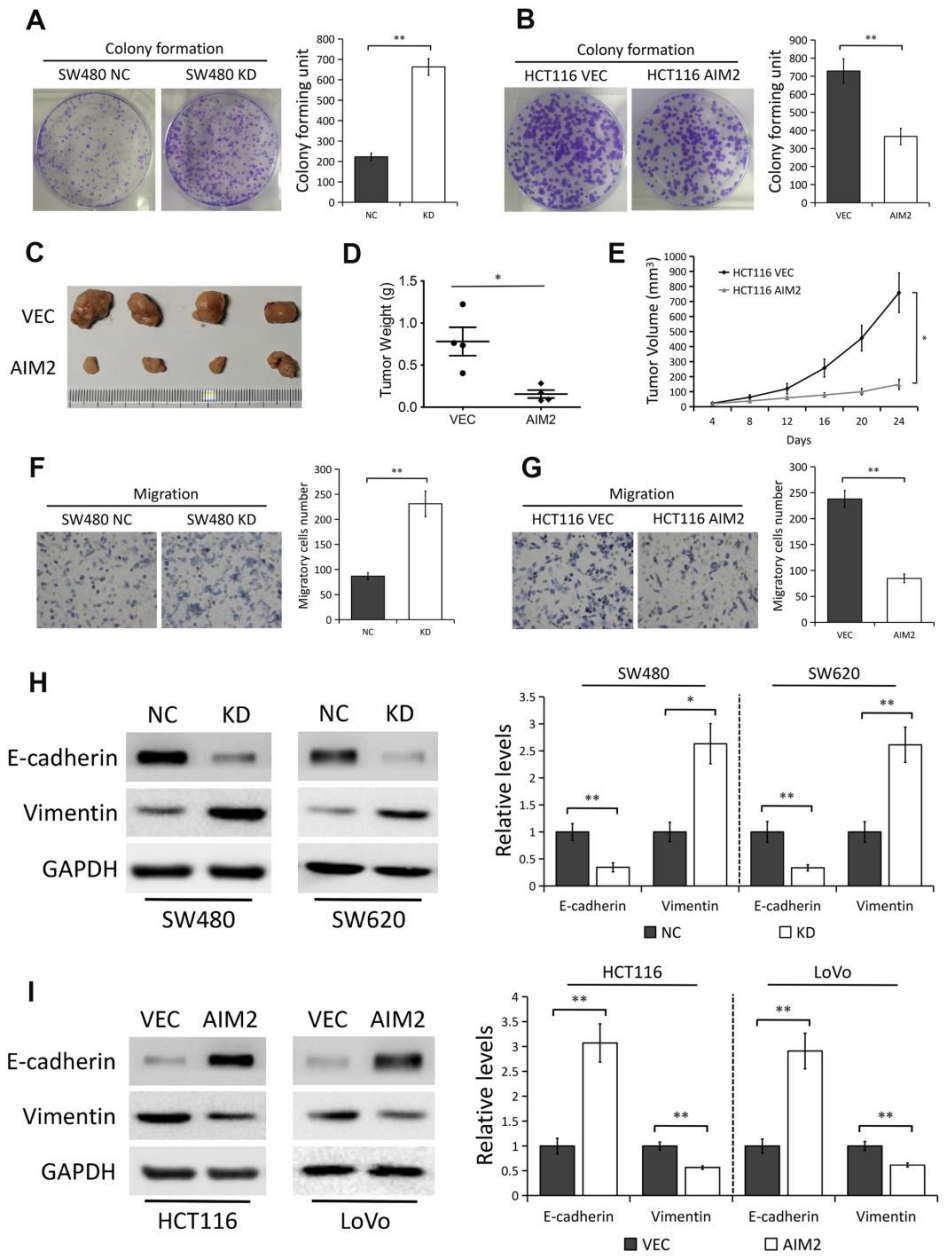
**AIM2 plays anti-carcinogenic roles in CRC.** (**A**) Colony formation assays to test viability of SW480 cells stably transfected with control-shRNA (NC) or shRNA against AIM2 (KD). Quantitative analysis results were presented as the mean±SEM (n=3). (**B**) Colony formation assays to test viability of HCT116 cells stably transfected with empty vector (VEC) or plasmid encoding human AIM2 (AIM2). Quantitative analysis results were presented as the mean±SEM (n=3). (**C**) Subcutaneous xenograft tumor growth in nude mice (4 per group) was measured and compared in HCT116 (VEC vs. AIM2) cell lines, and the representative image of tumors was shown. (**D**) Scatter plot analysis of tumor weight of each group was presented. (**E**) The volumes of the tumors measured every 4 days during the indicated period were shown. (**F**) Transwell assays to test migration ability of SW480 NC and KD cells. Quantitative analysis results were presented as the mean±SEM (n=3). (**G**) Transwell assays to test migration ability of HCT116 VEC and AIM2 cells. Quantitative analysis results were presented as the mean±SEM (n=3). (**H**) Western blots of E-cadherin and Vimentin protein in SW480 and SW620 cells stably transfected with control-shRNA (NC) or shRNA against AIM2 (KD). GAPDH as a loading control. Each experiment was performed at least triplicate and the bands were quantified and presented as the mean±SEM. (**I**) Western blots of E-cadherin and Vimentin protein in HCT116 and LoVo cells stably transfected with empty vector (VEC) or plasmid encoding human AIM2 (AIM2). GAPDH as a loading control. Each experiment was performed at least triplicate and the bands were quantified and presented as the mean±SEM. *P<0.05, **P<0.01, based on a two-tailed paired Student’s t-test.

Considering AIM2 expression was greatly decreased in CRC tissues and reduced AIM2 in CRC correlated with tumor size, depth of invasion, LNM and TNM stage, we thus examined the potential oncogenic roles of AIM2 in CRC cells. Colony formation analysis showed that AIM2 knockdown in CRC cells significantly enhanced cell proliferation ability (P<0.01, Figure 3A and Supplementary Figure 2B), while ectopic AIM2 expression had the opposite effect in HCT116 cells (P<0.01, Figure 3B). In light of our *in vitro* findings, we tested the function of AIM2 in colorectal tumorigenesis with a nude mouse xenograft model. HCT116 cells stably expressing AIM2 (AIM2) or empty vector (VEC) were implanted onto the subcutaneous sites of nude mice. Growth of the implanted tumors was monitored and tumor sizes were measured every 4 days (Figure 3C). Results showed that tumor weights (Figure 3D) and sizes (Figure 3E) derived from AIM2-overexpressed HCT116 cells were notably reduced compared to their negative controls, suggesting the inhibitory effect of AIM2 on colorectal tumor growth.

Next, we evaluated the effect of AIM2 on cell migration. Results from transwell assay showed that the migration ability of CRC cells was greatly enhanced when cells lacked AIM2 (P<0.01, Figure 3F and Supplementary Figure 2C). Inversely, HCT116 cells stably expressing AIM2 showed a significant decrease of migratory cells as compared with their negative controls (P<0.01, Figure 3G). To further explore the effect of AIM2 on tumor metastasis, we examined the protein markers of EMT progress, which is an early event in the metastasis of cancer [24, 25]. In AIM2-depleted SW480 and SW620 cells, the protein expression of epithelial marker E-cadherin was greatly decreased, while the mesenchymal marker Vimentin protein level was significantly increased (Figure 3H). On the contrary, AIM2 overexpression in HCT116 and LoVo cells markedly promoted E-cadherin expression, but inhibited Vimentin expression (Figure 3I).

### AIM2 inhibits Gli1 expression through SMO-independent pathway in CRC

**Figure 4:**
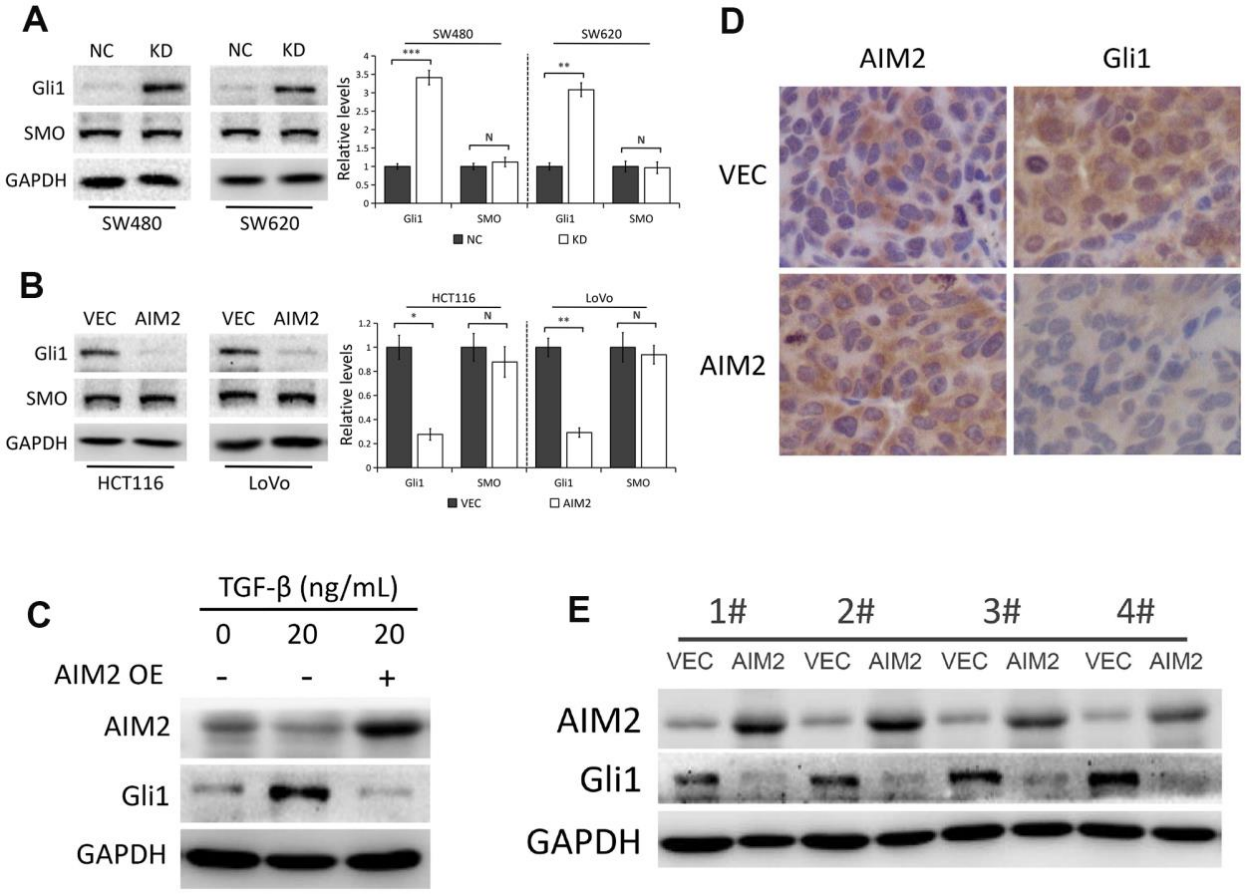
**AIM2 inhibits Gli1 expression independent of SMO.** (**A**) Western blots of Gli1 and SMO protein expression in SW480 and SW620 cells stably transfected with control-shRNA (NC) or shRNA against AIM2 (KD). GAPDH as a loading control. Each experiment was performed at least triplicate and the bands were quantified and presented as the mean±SEM. (**B**) Western blots of Gli1 and SMO protein expression in HCT116 and LoVo cells stably transfected with empty vector (VEC) or plasmid encoding human AIM2 (AIM2). GAPDH as a loading control. Each experiment was performed at least triplicate and the bands were quantified and presented as the mean±SEM. (**C**) Western blots of AIM2 and Gli1 protein expression in HCT116 cells treated with 20 ng/mL TGF-β for 48 h. (**D**) Representative images of IHC staining of AIM2 and Gli1 in subcutaneous tumors derived from HCT116 (VEC vs. AIM2) cells. (**E**) Western blots of AIM2 and Gli1 protein expression in subcutaneous tumors. N, nonsignificant, *P<0.05, **P<0.01, ***P<0.001, based on a two-tailed paired Student’s t-test.

Numerous studies have demonstrated that aberrant Gli1 expression in Hedgehog pathway plays a critical role in the development of CRC [20, 26] and Gli1 promotes EMT in CRC cells [27, 28]. Thus, we speculated whether Gli1 is regulated by AIM2 in CRC cells. Interestingly, Gli1 protein levels were markedly increased in AIM2-depleted SW480 and SW620 cells compared to the negative control (Figure 4A). Conversely, AIM2 elevation notably impaired Gli1 protein expression in HCT116 and LoVo cells (Figure 4B). In accordance with previous studies [29, 30], elevated Gli1 protein expression occurred in HCT116 cells treated with 20 ng/mL TGF-β for 48 h (Figure 4C). Interestingly, the increased Gli1 protein expression induced by TGF-β treatment was reversed when cells overexpressed AIM2 (Figure 4C), indicating that AIM2 overexpression impaired the effect of TGF-β on Gli1 expression. In addition, AIM2 regulation of Gli1 was also examined in xenografted tumors. Results of IHC (Figure 4D) and Western blots (Figure 4E) showed a remarkably decreased expression of Gli1 protein in tumors derived from HCT116 cells stably expressing AIM2, suggesting Gli1 may be involved in tumor growth regulated by AIM2 in CRC.

SMO, as the central signal transducer in the classical Hh pathway, activates Gli1 by blocking its inhibitory partner SUFU [22]. To investigate whether AIM2 inhibits Gli1 expression through SMO-dependent pathway, we used Western blot analysis to test SMO protein expression by AIM2 genetic manipulation in CRC cells. As shown in Figure 4A, 4B, AIM2 knockdown or overexpression had minimal effect on SMO protein level, indicating that there might be other molecules or signaling involved in the regulation of Gli1 expression by AIM2 in CRC cells.

### AIM2 suppresses CRC cell proliferation and migration via regulating Gli1

**Figure 5:**
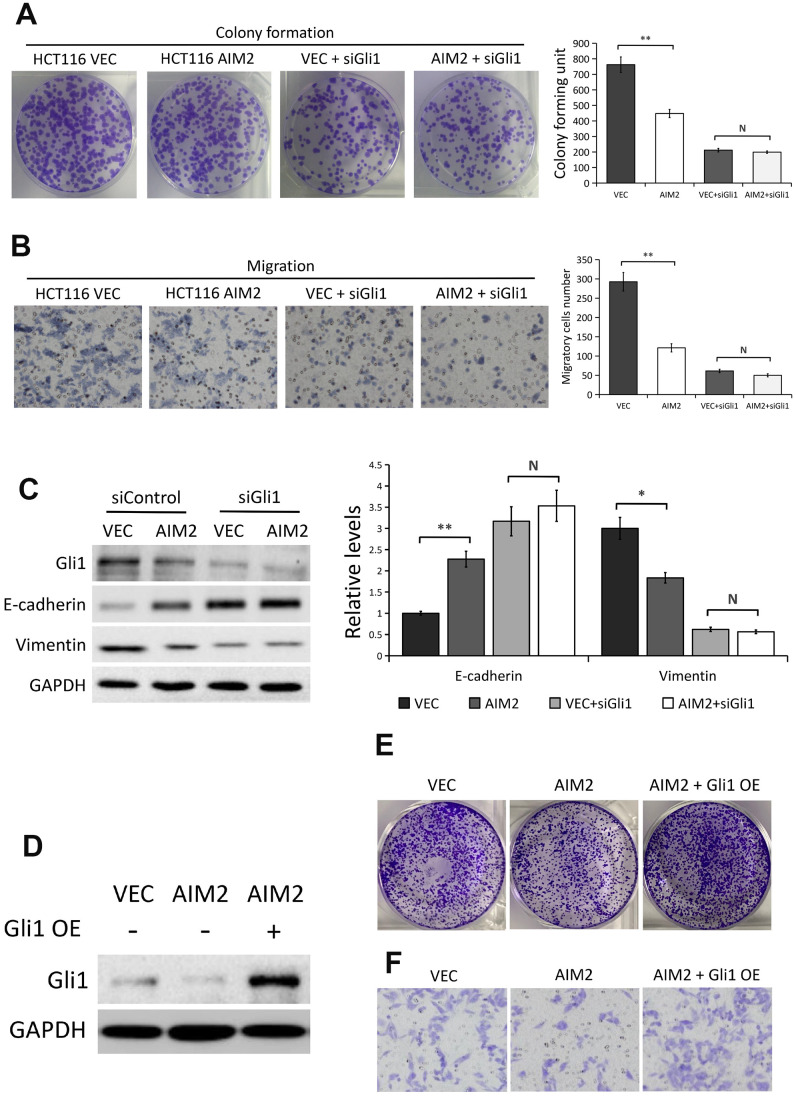
**AIM2 inhibits HCT116 cell proliferation, migration and EMT progress in a Gli1-dependent manner.** (**A**) Colony formation assays to test viability of HCT116 cells stably transfected with empty vector (VEC) or plasmid encoding human AIM2 (AIM2) with or without Gli1 siRNA treatment. Quantitative analysis results were presented as the mean±SEM (n=3). (**B**) Transwell assays to test migration ability of HCT116 cells stably transfected with empty vector (VEC) or plasmid encoding human AIM2 (AIM2) with or without Gli1 siRNA treatment. Quantitative analysis results were presented as the mean±SEM (n=3). (**C**) Western blots of E-cadherin and Vimentin protein expression in HCT116 cells stably transfected with empty vector (VEC) or plasmid encoding human AIM2 (AIM2) in the presence or absence of Gli1 siRNA. GAPDH as a loading control. Each experiment was performed at least triplicate and the bands were quantified and presented as the mean±SEM. (**D**) Western blots of Gli1 protein expression in LoVo (VEC vs. AIM2) cells transfected with plasmids encoding human Gli1 or empty vector. (**E, F**) Colony formation assay (**E**) and transwell assay in LoVo (VEC vs. AIM2) cells transfected with plasmids encoding human Gli1 or empty vector. N, nonsignificant, *P<0.05, **P<0.01, based on a two-tailed paired Student’s t-test or ANOVA.

To detect whether Gli1 is implicated in the regulation of AIM2 on CRC cell proliferation and migration ability, we used Gli1 specific siRNA to silence Gli1 expression in HCT116 cells with/without AIM2 overexpression. Results from colony formation assays showed that the proliferation ability of HCT116 cells was greatly impaired when AIM2 was overexpressed (P<0.01, Figure 5A), whereas there was no significant effect of AIM2 overexpression on cell proliferation property when cells lacked Gli1 (P>0.05, Figure 5A). Next, we conducted transwell migration assays to evaluate the effect of AIM2 on cell migration in the absence of Gli1. As shown in Figure 5B, the migratory HCT116 cells was markedly reduced when cells overexpressed AIM2 (P<0.01), but ectopic AIM2 failed to inhibit cell migration in Gli1-silenced HCT116 cells (P>0.05). Consistent with these results, forced expression of AIM2 potently enhanced E-cadherin expression but suppressed Vimentin expression (Figure 5C). However, ectopic AIM2-induced E-cadherin increases and Vimentin losses were reversed in Gli1-depleted HCT116 cells (Figure 5C).

Furthermore, we used plasmids encoding human Gli1 to overexpress Gli1 in AIM2-overexpressed LoVo cells and the overexpression efficiency was confirmed by Western blot analysis (Figure 5D). Colony formation assays and migration assays showed that the decreased proliferation and migration ability induced by AIM2 overexpression was reversed after transfection with plasmids encoding human Gli1 (Figure 5E, 5F). Collectively, these above data suggested that AIM2 regulates CRC cell proliferation and migration in a Gli1-dependent pathway.

### AIM2 regulates Gli1 expression and EMT progress through AKT/mTOR pathway

It has been reported that AIM2 regulates CRC cell viability via AKT pathway [15–17] and depletion of AIM2 expression promotes hepatocarcinoma progression through mTOR activation [13]. Our present study suggests the possibility that other molecules or signaling might be involved in the regulation of Gli1 expression by AIM2 independent of SMO in CRC cells. Considering that an activated AKT/mTOR pathway was reported to promote Gli1 activity in a SMO-independent manner [22], we thus raised a presumption that AKT/mTOR pathway might be implicated in AIM2-induced Gli1 inhibition.

**Figure 6:**
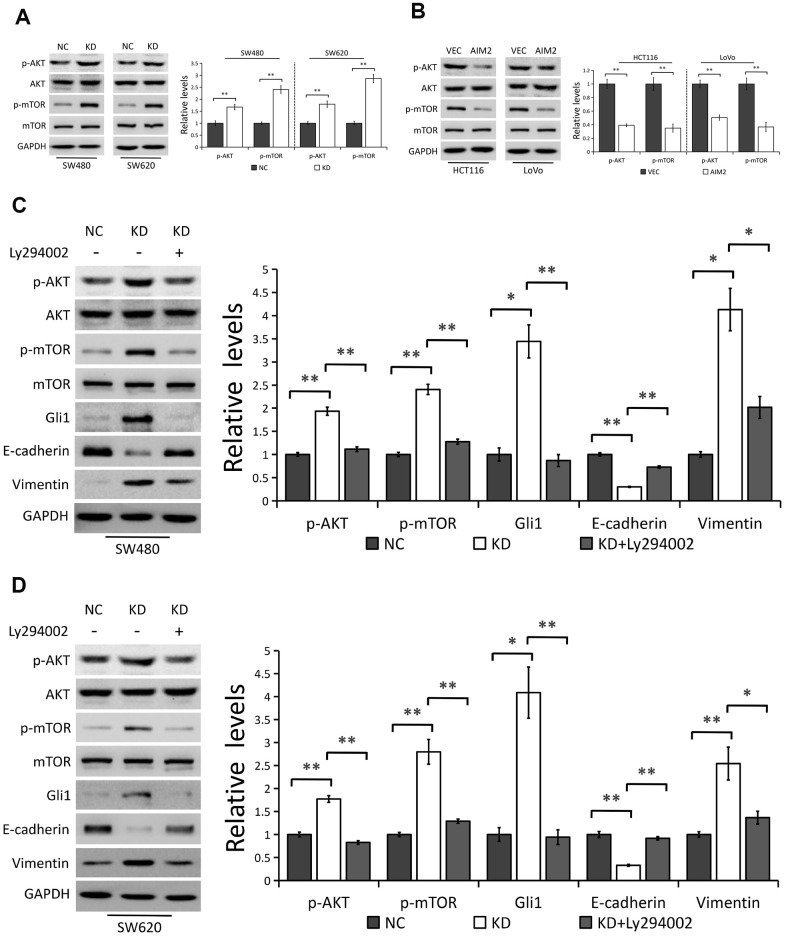
**AIM2 regulates Gli1-mediated EMT through AKT/mTOR pathway.** (**A**) Western blots of indicated proteins in SW480 and SW620 cells stably transfected with control-shRNA (NC) or shRNA against AIM2 (KD). GAPDH as a loading control. Each experiment was performed at least triplicate and the bands were quantified and presented as the mean±SEM. (**B**) Western blots of indicated proteins in HCT116 and LoVo cells stably transfected with empty vector (VEC) or plasmid encoding human AIM2 (AIM2). GAPDH as a loading control. Each experiment was performed at least triplicate and the bands were quantified and presented as the mean±SEM. (**C**, **D**) Western blots of indicated proteins in SW480 (**C**) and SW620 (**D**) cells stably transfected with control-shRNA (NC) or shRNA against AIM2 (KD) with or without Ly294002 (20 μM). GAPDH as a loading control. Each experiment was performed at least triplicate and the bands were quantified and presented as the mean±SEM. *P<0.05, **P<0.01, based on a two-tailed paired Student’s t-test or ANOVA.

In line with previous studies [13, 15–17], we further confirmed the inhibitory effect of AIM2 on AKT/mTOR signaling pathway. As shown in Figure 6A, 6B, depleted expression of AIM2 notably increased the levels of phosphorylated AKT (p-AKT) and phosphorylated mTOR (p-mTOR) in both SW480 and SW620 cells, with no effect on their total protein levels. Conversely, Western blot analysis also identified lower phosphorylation levels of AKT and mTOR in AIM2-overexppressed HCT116 and LoVo cells than the negative controls. We next treated AIM2-deficiency cells with Ly294002, an indirect inhibitor of AKT, to examine the role of AKT/mTOR signaling pathway in AIM2-mediated Gli1 suppression. Results showed that Ly294002 incubation remarkably suppressed AKT phosphorylation, followed by subsequent mTOR inhibition in AIM2-depleted SW480 and SW620 cells (Figure 6C, 6D). We also found that increased Gli1 protein expression induced by AIM2 knockdown was reversed in cells with Ly294002 treatment (Figure 6C, 6D), indicating the involvement of AKT/mTOR pathway in AIM2-regulated Gli1 inhibition. In addition, AIM2-deficency-mediated EMT progress was also reversed in cells incubated with Ly294002 as evidenced by the results that Ly294002 treatment significantly increased E-cadherin expression and suppressed Vimentin expression in AIM2-depleted SW480 and SW620 cells (Figure 6C, 6D).

Collectively, our results indicated that AKT/mTOR pathway is indispensable for AIM2-regulated Gli1 inhibition and EMT progress in CRC cells.

## DISCUSSION

The innate immune DNA sensor AIM2 was originally isolated from healthy melanocytes [6]. AIM2 directly binds dsDNA and initiates the recruitment of ASC and caspase-1 to activate the inflammasome [7, 8]. In cancers, the role of AIM2 is contentious and it could be either oncogenic or tumor suppressive. AIM2 protein levels were reported to be frequently overexpressed in several types of malignancies including OSCC and on-small cell lung cancer, and its overexpression promoted cancer progression and predicted poor survival of patients [9–11]. On the other hand, loss of AIM2 expression was observed in multiple tumor types including CRC [12–18]. In CRC, reduced AIM2 expression exhibited oncogenic properties and was closely associated with poor outcome [15–18]. However, the concrete molecular mechanisms of AIM2 in CRC development are elusive and remain to be further explored.

In the present work, we provide evidence that AIM2 was commonly downregulated in human CRC and was further reduced in those with LNM. Moreover, clinical data analysis showed that loss of AIM2 significantly correlated with tumor size, depth of invasion, LNM and TNM stage in patients suffering from CRC. On functional verification, our loss-of-function and gain-of-function experiments *in vitro* and *in vivo* suggested a tumor-suppressive role of AIM2 in CRC development. A published previous study [31] demonstrated that AIM2 enhanced invasion of HCT116 CRC cells, and they also found that Vimentin mRNA expression was increased, whereas E-cadherin mRNA expression was decreased in AIM2-overexpressed HCT116 cells, which conflicts with our findings. Our results showed that AIM2 inhibited CRC cell migration and reversed EMT progress as evidenced by the fact that disruption of AIM2 inhibited E-cadherin expression and promoted Vimentin expression while AIM2 overexpression did the opposite in CRC cells, which is consistent with the previous study [17].

The Hedgehog (Hh) signaling pathway plays an essential role in cancer development and progression [19, 20]. Gli1, as a key transcriptional factor of Hh pathway, has been proposed as a candidate oncogene in CRC [20, 26, 27]. In the present study, we found that AIM2 regulated Gli1 expression in CRC cells, which is evidenced by the following observations. Our *in vitro* experiments showed that Gli1 protein levels were markedly increased in AIM2-depleted cells, but notably decreased in cells with AIM2 overexpression. In addition, AIM2 regulation of Gli1 was also examined in xenografted tumors. IHC and Western blot results showed a remarkably decreased expression of Gli1 protein in tumors derived from HCT116 cells stably expressing AIM2.

To investigate whether Gli1 is responsible for AIM2 regulation of CRC cell proliferation and migration, we silenced Gli1 in HCT116 cells with/without AIM2 overexpression. Results showed that AIM2 overexpression impaired cell proliferation and migration ability and suppressed EMT progress which was reversed by Gli1 depletion. In addition, we found that the decreased proliferation and migration ability induced by AIM2 overexpression was reversed after transfection with plasmids encoding human Gli1 (Figure 5E, 5F), which indicated the anti-carcinogenic roles of AIM2 in CRC cells is mediated by Gli1.

SMO, as the central signal transducer in the classical Hh pathway, activates Gli1 by blocking its inhibitory partner SUFU [22]. However, AIM2-induced Gli1 suppression in CRC cells is unlikely through the classical Hh pathway since SMO protein expression was not affected by AIM2 genetic manipulation, indicating that there might be other molecules or signaling involved in the regulation of Gli1 by AIM2 in CRC cells.

Previous studies showed that AIM2 regulates CRC cell viability via AKT pathway [15–17] and an activated AKT/mTOR pathway promotes Gli1 activity in a SMO-independent manner [22]. In the present work, we found that AKT/mTOR pathway is implicated in AIM2-regulated Gli1 inhibition and EMT progress in CRC cells as evidenced by the fact that AIM2 depletion increased Gli1 protein expression and promoted EMT progress which was reversed by treatment of Ly294002, an indirect inhibitor of AKT.

In conclusion, we found that AIM2 expression was significantly decreased in CRC tissues and loss of AIM2 was strongly correlated with malignant properties of CRC cells. Mechanically, the tumor-suppressive functions of AIM2 in CRC were mediated through its participation in repressing the AKT/mTOR pathway, and the inactivated AKT/mTOR failed to increase Gli1 protein expression, resulting in the inhibition of cell proliferation and migration. This may highlight a new entry point for treating CRC by targeting the AIM2/AKT/mTOR/Gli1 signaling axis.

## MATERIALS AND METHODS

### CRC tissues and cell lines

Fresh and formalin-fixed tissue samples were collected from patients who underwent surgical resection in the First Affliated Hospital of Wannan Medical College from January 2015 to January 2018. The clinicopathological features of these patients are shown in Table 1. The Institute Research Medical Ethics Committee of the First Affliated Hospital of Wannan Medical College granted approval for this study, which was conducted in compliance with the Declaration of Helsinki. Written informed consent was obtained from all patients.

Five human CRC cell lines (HCT116, CCL244, SW480, SW620 and LoVo) were obtained from the Cell Bank of the Chinese Academy of Sciences, Shanghai, China. Cells were routinely cultured and incubated in RPMI 1640 medium (Gibco; Thermo Fisher Scientific, Inc., Waltham, MA, USA) supplemented with 10% fetal bovine serum (FBS; Gibco; Thermo Fisher Scientific, Inc., Waltham, MA, USA) and 1% penicillin/streptomycin (P/S; Gibco; Thermo Fisher Scientific, Inc., Waltham, MA, USA) at 37° C in a humidified atmosphere containing 5% CO_2_.

### Immunohistochemistry (IHC)

IHC staining of paraffin-embedded human or mice tumor samples were conducted according to the manufacturer’s instructions. Samples were deparaffinized, rehydrated, subjected to antigen retrieval, and blocked with 3% hydrogen dioxide, followed by incubating with the primary antibodies recognizing human AIM2 (dilution 1:200; #ab93015, Abcam) or human Gli1 (dilution 1:200; #ab15179, Abcam) overnight at 4° C. Next day, the sections were incubated with secondary antibody and were visualized using a tissue staining kit (Zhongshan Biotechnology, Beijing, China). Staining intensity was classified as 0 (lack of staining), 1 (mild staining), 2 (moderate staining) or 3 (strong staining); staining percentage was designated as 1 (<25%), 2 (25%-50%), 3 (51%-75%), or4 (>75%). The final staining score was calculated by the multiple of color intensity and positive cell percentage, which ranged from 0 to 12. Scores 0-4 were described as none or low expression, while 5-12 as high expression.

### Protein extraction and western blot analysis

Cells or tumor tissues were lysed in cold RIPA buffer (Beyotime, Beijing, China) supplemented with protease inhibitors (Roche, CA, USA) and run on SDS-PAGE gels. After transferring to the PVDF membranes, immunoblots were analyzed using the primary antibodies at 4° C overnight and were incubated with fluorescent secondary antibodies for 1 hour. Then the proteins were visualized by chemiluminescence. Antibodies used in this study were listed in Table 2.

**Table 2:** Antibody information.

**Name**	**Company**	**Catalog number**	**Dilution**
AIM2	Abcam	ab93015	1:1000
p-AKT (Ser473)	Cell Signaling Technology	4058	1:1000
AKT	Cell Signaling Technology	9272	1:1000
p-mTOR	Cell Signaling Technology	9205	1:1000
mTOR	Cell Signaling Technology	2983	1:1000
E-cadherin	Cell Signaling Technology	3195	1:1000
Vimentin	Cell Signaling Technology	5741	1:1000
Gli1	Abcam	ab151796	1:1000
SMO	Abcam	ab38686	1:1000
GAPDH	Beyotime	AG019	1:1000

### Stable cell lines and small interfering RNA (siRNA) transfection

Lentiviral vectors plasmids were constructed by GENECHEM Biotech at Shanghai, China. Transfection procedures were performed according to the manufacturer’s instructions. Briefly, cells were transduced with lentiviruses for 10 h and were selected with puromycin (4 μg/ml) for 7-10 days. Transfection of siRNAs targeting human AIM2 and Gli1 was performed with Lipofectamine^TM^ RNAiMax (Invitrogen) at a final concentration of 20 nM according to the manufacturer’s instructions. The sequences specific for human AIM2 are 5′-CCCGAAGATCAACACGCTTCA-3′ and 5′-GGAGAAAGUUGAUAAGCAA-3′. The sequence specific for human Gli1 5′-CUCCACAGGCAUACAGGAU-3′.

### Cell viability and migration assay

For the colony formation assays, about 1,000 cells were cultivated in 6-well plates for 7-10 days. Colonies formed were washed with PBS, fixed with 4% paraformaldehyde (Beyotime, Beijing, China) and stained with 0.1% crystal violet (Beyotime, Beijing, China). Colonies containing more than 50 cells for each well were counted.

For transwell migration assays, cells were seeded into the upper chambers (Corning Incorporated, USA) in serum-free RPMI 1640 and the lower chamber was filled with RPMI 1640 containing 10% FBS. After incubation for 12-48 h, cells which had migrated through the membrane were fixed with 4% paraformaldehyde (Beyotime, Beijing, China) and stained with 0.1% crystal violet.

### Subcutaneous xenograft

Nude mice (BALB/c, SPF grade, 16-18 g, 4-5 weeks and male) were purchased from Shanghai SLRC Laboratory Animal Co., Ltd. Nude mice were injected subcutaneously with 5×10^6^ CRC cells. Body weight and tumor sizes were measured every 4 days. At the endpoint, tumors were harvested, weighted and stored for further analysis. All animal experimental procedures were approved by the Animal Ethics Committee of Wannan Medical College (Wuhu, China).

### Statistical analysis

The results were shown as mean ± SEM. A two-tailed Student t-test was used to compare the variables of two groups and one-way analysis of variance (ANOVA) was used for the comparison of multiple groups. Analysis of IHC results was performed by chi-square statistical test or Fisher’s exact test. A P value of <0.05 was considered as significant.

## Supplementary Material

Supplementary Figures
