# Supplementary material for: AIM2 inhibits colorectal cancer cell proliferation and migration through suppression of Gli1
Source: Aging (Albany NY). 2020 Dec 3;13(1):1017–31. doi: 10.18632/aging.202226 (PMC7835022; doi:10.18632/aging.202226)
Supplement: Supplementary Figures [file aging-13-202226-s001.pdf]

SUPPLEMENTARY FIGURES

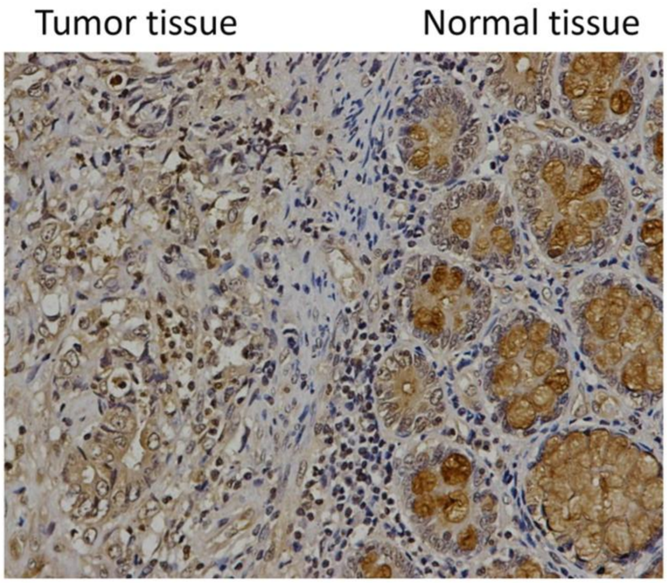

Supplementary Figure 1. IHC staining of AIM2 in CRC tumor tissues and surrounding normal tissues from the same slide.

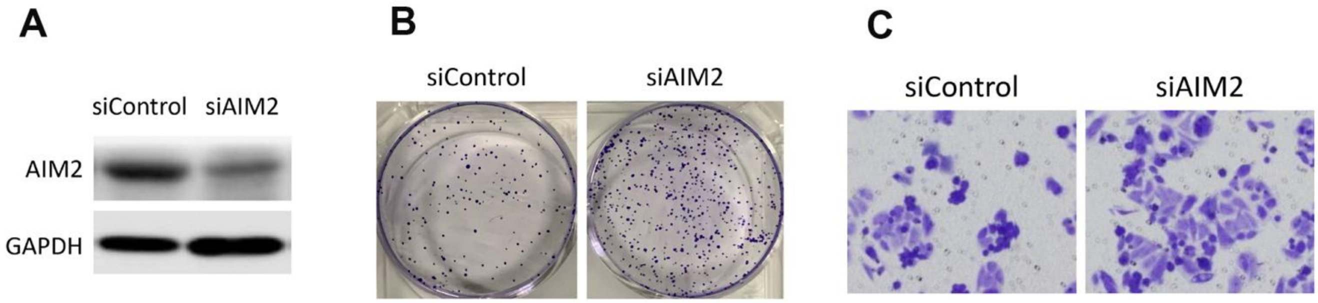

Supplementary Figure 2. AIM2 plays anti-carcinogenic roles in CRC. (**A**) Western blots of AIM2 protein in SW620 cells transfected with control-siRNA (siControl) or siRNA against AIM2 (siAIM2). GAPDH as a loading control. (**B**) Colony formation assays to test proliferation ability of SW620 cells transfected with siControl or siAIM2. (**C**) Transwell assays to test migration ability of SW620 cells transfected with siControl or siAIM2.
